# Personalized Perioperative Music Listening: A Human-Centered Approach to Precision Anesthesia

**DOI:** 10.7759/cureus.113073

**Published:** 2026-07-21

**Authors:** Michael Egbujionuma

**Affiliations:** 1 Anesthesia, Kaiser Permanente, Walnut Creek, USA

**Keywords:** anxiolytic premedication, emergence delirium, music listening, narrative review, nurse anesthesia, patient-centered anesthesia, perioperative music listening intervention, precision anesthesia, preoperative anxiety

## Abstract

Modern anesthesia has achieved remarkable advances in safety, monitoring, and pharmacologic precision, yet perioperative anxiety continues to be a common and under-addressed aspect of surgical care, affecting a substantial proportion of patients undergoing surgery. Patients increasingly request personalized music during transport to the operating room and prior to anesthetic induction. Although often viewed as a comfort measure, this practice may represent an important component of a wider movement toward human-centered perioperative care. This narrative review summarizes contemporary evidence on perioperative music listening interventions and proposes a framework for integrating individualized sensory preferences into anesthesia practice. Sixteen empirical studies, including major systematic reviews, meta-analyses, and randomized controlled trials, are synthesized across general surgery, cardiac surgery, obstetric anesthesia, intensive care, awake craniotomy, orthopedic and breast cancer surgery, and pediatric anesthesia. The evidence generally demonstrates reductions in preoperative anxiety, postoperative pain, analgesic consumption, sedative requirements, and selected physiologic stress responses, with one randomized trial reporting greater anxiety reduction with music than with oral midazolam premedication. A neurochemical mechanism involving dopaminergic reward circuitry, hypothalamic-pituitary-adrenal axis modulation, immune mediators, and oxytocinergic social-affiliation pathways is also reviewed. A practical perioperative workflow, comparison with prior reviews, and a research agenda focused on personalization and digital perioperative profiling are presented. Personalized perioperative music listening is a low-cost, scalable, evidence-supported intervention that may help anesthesia evolve toward a more humane, patient-centered surgical experience.

## Introduction and background

Anesthesia practice has traditionally been organized around physiologic stability, pharmacologic precision, and procedural effectiveness. Advances in monitoring, airway management, anesthetic pharmacology, and perioperative safety systems have dramatically reduced anesthesia-related morbidity and mortality over recent decades. In spite of these achievements, many surgical patients continue to experience significant emotional distress, fear, and anxiety in the moments preceding induction of anesthesia. A 2022 review estimated that preoperative anxiety affects approximately 48% of surgical patients in pooled international analyses, with elevated rates among younger patients, female patients, and those with limited prior surgical exposure [1].

For many patients, the interval between entering the operating room and losing consciousness is among the most psychologically vulnerable periods of the surgical experience. During this brief window, patients encounter unfamiliar equipment, bright lights, alarms, conversations between strangers, and a perceived loss of personal control. In observational studies, these experiences have been associated with increased sympathetic activation, greater preoperative anxiety, increased postoperative pain perception, delayed recovery, and reduced patient satisfaction; such data demonstrate association rather than established causation, and the relationship between anxiety and downstream outcomes is likely bidirectional and multifactorial [1,2].

A request that anesthesia clinicians increasingly encounter from patients and their families is to play personalized music during the immediate preinduction period. Although formal epidemiologic data quantifying the frequency of such requests remain limited, the high patient satisfaction and near-universal endorsement of perioperative music documented in the randomized trials discussed below are consistent with strong patient receptivity to this intervention. What may seem at first to be a minor comfort intervention may, in fact, represent an emerging shift toward patient-centered anesthesia care. In this review, precision anesthesia refers to the individualization of perioperative care to the specific physiologic, pharmacologic, psychological, and sensory characteristics of each patient; the term is used as a conceptual framework rather than as a synonym for pharmacogenomic precision medicine. This review examines the evidence supporting perioperative music-listening interventions, situates personalized music selection within this deliberately broadened conceptual view of precision anesthesia, presents a summary of included studies and a comparison with prior reviews, and proposes a practical workflow and research agenda for incorporating individualized emotional and sensory care into routine anesthetic practice.

## Review

Methodology

This article is a narrative review and is not designed or reported as a systematic review or meta-analysis; the Preferred Reporting Items for Systematic Reviews and Meta-Analyses (PRISMA) framework and a PRISMA flow diagram therefore do not apply. The aim is to synthesize representative high-quality evidence on perioperative music interventions and to translate that evidence into a clinical framework relevant to anesthesia practice.

PubMed, the Cochrane Database of Systematic Reviews, and the Web of Science Core Collection were searched from database inception through March 31, 2026. The search combined controlled vocabulary and free-text terms using the Boolean structure: (music OR “music listening” OR “music intervention” OR “music therapy” OR “music medicine”) AND (perioperative OR preoperative OR intraoperative OR postoperative OR anesthesia OR surgery) AND (“preoperative anxiety” OR “perioperative anxiety” OR “postoperative pain” OR sedation OR “emergence delirium” OR “patient satisfaction” OR “precision anesthesia”). Searches were limited to English-language, peer-reviewed publications involving human participants.

Eligibility favored systematic reviews, meta-analyses, and randomized controlled trials, while selected mechanistic and qualitative studies were included where they added explanatory value. Titles and abstracts were screened for relevance to perioperative music interventions and anesthesia-relevant outcomes, and potentially relevant full texts were then reviewed. Reference lists of relevant reviews and randomized controlled trials were manually screened for additional eligible studies (backward citation searching). Studies in surgical, obstetric, critical-care, and pediatric anesthesia were included and were appraised narratively for relevance, study design, and contribution to the proposed clinical framework.

Consistent with a narrative rather than systematic design, records were not tracked at the level of a formal count of the numbers identified, screened, and excluded, and a formal quantitative risk-of-bias assessment (for example, the Cochrane Risk of Bias 2 tool for randomized trials, Risk Of Bias In Non-randomized Studies - of Interventions (ROBINS-I) for non-randomized studies, or Grading of Recommendations Assessment, Development and Evaluation (GRADE) for certainty of evidence) was not performed. These methodical choices are acknowledged as limitations and are discussed further below. The synthesized evidence comprises 14 empirical studies (systematic reviews, meta-analyses, and randomized controlled trials), one mechanistic review, and one published trial protocol, 16 sources in total. Where reported, anxiety and related outcomes were most commonly quantified using validated instruments, including the State-Trait Anxiety Inventory (STAI) [3], the Visual Analog Scale for Anxiety (VAS-A) [4], the Amsterdam Preoperative Anxiety and Information Scale [5], Pain Catastrophizing Scale [6], and standardized measures of analgesic and sedative consumption.

The psychological landscape of the operating room

The operating room is an inherently stressful environment. According to observational and cohort data, preoperative anxiety has been associated with increased catecholamine release, elevated heart rate and blood pressure, increased intraoperative anesthetic requirements, increased postoperative pain, prolonged recovery, and reduced patient satisfaction; these associations do not, on their own, establish that anxiety directly causes each downstream outcome [1,2]. Traditional strategies for reducing preoperative anxiety include benzodiazepine premedication, verbal reassurance, family presence, and structured preoperative education. Even though effective for many patients, these strategies do not consistently address the emotional or sensory dimensions of individualized comfort, and routine benzodiazepine use has declined in many institutions because of postoperative delirium concerns, notably in older adults [1]. Music engages emotional, cognitive, autonomic, and neurochemical pathways simultaneously, and unlike pharmacologic anxiolysis, music listening is noninvasive, low cost, easily implemented, scalable across clinical environments, and associated with minimal risk [7,8].

Neurobiological basis of music's perioperative effects

A key review by Chanda and Levitin synthesized evidence that music engages four major neurochemical systems relevant to the perioperative period: reward, motivation, and pleasure mediated by dopaminergic and endogenous opioid signaling; stress and arousal mediated by the hypothalamic-pituitary-adrenal axis, reduced cortisol, and autonomic modulation; immune function via effects on immunoglobulin A and cytokines; and social affiliation via oxytocinergic pathways [8]. These mechanisms together present a plausible biological basis for the anxiolytic, analgesic, and stress-attenuating effects of perioperative music observed in clinical trials [7,8]. Importantly, this neurochemical engagement does not require active musical performance; passive music listening alone is sufficient to produce measurable effects [8].

A widely used distinction in the music-and-health literature separates music therapy, in which a credentialed music therapist delivers an individualized therapeutic process, from music medicine, in which medical personnel offer passive listening to pre-recorded music [2]. Most perioperative interventions described in surgical and anesthesia trials, including the present clinical recommendations, fall within the music medicine category. The distinction is important when interpreting effect sizes and when designing institutional programs, because credentialed music therapy is a more resource-intensive intervention than perioperative music listening.

Evidence supporting perioperative music listening interventions

A considerable body of literature supports music listening interventions in perioperative medicine. The 2013 Cochrane systematic review by Bradt and colleagues pooled 26 randomized trials enrolling 2,051 surgical patients and concluded that music listening produced clinically meaningful reductions in preoperative anxiety, with a mean decrease of 5.7 points on the STAI compared with standard care [2]. The review also identified one large randomized trial in which preoperative music was more effective than oral midazolam for reducing anxiety, although the authors emphasized that this finding came from a single large study and should be interpreted with appropriate caution [2,9].

That trial by Bringman and colleagues randomized 372 elective surgical patients to oral midazolam (0.05-0.1 mg/kg) or relaxing music as premedication. The decline in state-anxiety scores was significantly greater in the music group than in the midazolam group (p<0.001; 95% CI for the between-group difference in anxiety decline, -3.8 to -1.8), with no detectable adverse effects [9]. Although replication is needed, this study supports the position that nonpharmacologic interventions deserve consideration alongside, and not solely as substitutes for, traditional anxiolytic premedication.

In 2015, Hole and colleagues published a landmark systematic review and meta-analysis in The Lancet that included 73 randomized controlled trials of perioperative music across several surgical populations [7]. Music significantly reduced postoperative pain (standardized mean difference (SMD) -0.77; 95% CI -0.99 to -0.56), anxiety (SMD -0.68; 95% CI -0.95 to -0.41), and analgesic consumption (SMD -0.37; 95% CI -0.54 to -0.20), and significantly increased patient satisfaction (SMD +1.09; 95% CI 0.51 to 1.68). Music remained effective even when patients were under general anesthesia, suggesting that auditory input continues to influence physiologic and affective pathways even in the absence of conscious awareness [7].

Cardiac Surgery

Kakar and colleagues evaluated 20 randomized trials of music in cardiac surgery and showed significant reductions in postoperative anxiety (SMD -0.50) and pain (SMD -0.51), with effects persisting for up to eight days postoperatively when multiple music sessions were provided [10]. These findings are clinically relevant in a population in which prolonged ventilation, ICU sedation, and high postoperative analgesic requirements are common.

Obstetric Anesthesia

Kakde and colleagues randomized 108 parturients undergoing elective cesarean delivery under spinal anesthesia to patient-selected music or control. Music listening was associated with a significant reduction in postoperative visual analog anxiety scores (mean difference -1.43) and in pain catastrophizing across rumination, magnification, and helplessness subscales; more than 95% of parturients reported good or excellent satisfaction with the intervention [11]. These results extend the perioperative music literature into obstetric anesthesia, a setting in which avoidance of systemic anxiolytics is frequently preferred for fetal safety reasons.

Critical Care and Mechanical Ventilation

In an intensive-care randomized trial of 36 mechanically ventilated, delirium-positive adults, Dalli and colleagues compared a music listening intervention with noise reduction and standard care. The music group showed significant reductions in delirium severity, pain, sedation requirements, and anxiety, as well as fewer days on mechanical ventilation compared with the other groups [12]. Although the sample is small, the findings suggest that music listening interventions may be relevant beyond the operating room itself, including in the immediate postoperative ICU period.

Brain Surgery and Awake Craniotomy

Preoperative anxiety is particularly common before brain surgery. A systematic review by Oteri and colleagues of 27 studies enrolling 2,558 brain-surgery patients found a preoperative anxiety prevalence ranging from 17% to 89%, with seven randomized controlled trials supporting the efficacy of music listening interventions, virtual reality, acupuncture, and pharmacologic strategies for anxiety reduction [13]. In the intraoperative setting, Jadavji-Mithani and colleagues studied 29 patients undergoing awake craniotomy who listened to major-key or minor-key musical pieces; patients consistently reported feeling more at ease and less anxious before, during, and after surgery, with no adverse reactions [14]. Awake neurosurgical procedures, in which patients must remain cooperative without heavy sedation, may be a particularly compelling indication for individualized music listening.

Orthopedic and Cancer Surgery

In a randomized controlled trial of 107 patients undergoing lower-limb orthopedic surgery under spinal anesthesia, Azi and colleagues found that patients who listened to instrumental music required significantly less intraoperative sedative medication (p=0.004) and reported reduced postoperative anxiety; nearly 99% endorsed the use of music during surgical procedures [15]. In breast cancer surgery, the systematic review by Tola and colleagues identified music listening interventions as having small-to-large effect sizes for reducing preoperative anxiety and large effect sizes for reducing postoperative pain [16].

Pediatric Anesthesia

Pediatric evidence is also encouraging. A systematic review and meta-analysis by van der Heijden and colleagues found statistically significant reductions in postoperative pain, anxiety, and distress in children receiving perioperative music listening interventions [17]. More recently, Muzzi and colleagues randomized 104 children undergoing adenotonsillectomy to intraoperative auditory stimulation with music, noise, ear inserts, or no intervention; music produced large effect sizes for reductions in pain on awakening and medium-to-large effect sizes for reductions in emergence delirium [18].

Synthesis Across Specialties

A 2024 meta-analysis by Li and colleagues pooled data from 19 randomized trials and found that perioperative music listening interventions significantly reduced postoperative pain and anxiety on the first postoperative day while attenuating fluctuations in blood pressure and heart rate [19]. An umbrella review by Aguero-Millan and colleagues, which evaluated 17 systematic reviews encompassing 188 controlled trials and 16,884 patients, identified music as the most studied and most consistently effective nonpharmacologic intervention for preoperative anxiety in adults [20]. Taken together, these reviews provide a converging body of evidence that perioperative music is associated with benefits across surgical specialties, age groups, anesthetic techniques, and outcome domains, although the observational and heterogeneous nature of much of the underlying data warrants prudent interpretation. It should be noted that several of these meta-analyses reported considerable statistical heterogeneity, reflecting variation in music type, delivery, timing, and outcome measurement across trials.

Patient-Selected Versus Standardized Music

While most published studies have evaluated generalized relaxing music or pre-curated playlists, an emerging line of research examines whether patient-selected music outperforms researcher-selected music. A published protocol by Petot and colleagues describes a randomized trial comparing self-selected music with predetermined music in gynecologic surgery, motivated by the hypothesis that personal meaning, familiarity, and cultural meaning may amplify the anxiolytic effect of music [21]. The relative superiority of patient-selected music remains an emerging hypothesis rather than an established finding, but if confirmed, it would have direct implications for how anesthesia clinicians design and implement perioperative music protocols.

Table 1 summarizes the empirical studies synthesized in this narrative review, including study type, population, intervention, and key findings.

**Table 1:** Summary of included studies RCT: randomized controlled trial; SR: systematic review; STAI: State-Trait Anxiety Inventory; VAS-A: Visual Analog Scale for Anxiety; VR: virtual reality

Author / Year (Citation)	Study type	Population (N)	Intervention	Key findings
Bradt et al., 2013 [2]	Cochrane SR & meta-analysis	26 RCTs; 2,051 adults	Music listening vs standard care, preoperative	Anxiety reduced ~5.7 STAI points; one RCT showed music was more effective than oral midazolam
Hole et al., 2015 [7]	SR & meta-analysis	73 RCTs; mixed surgery	Perioperative music (pre, intra, post)	Reduced pain (SMD -0.77), anxiety (-0.68), analgesic use (-0.37); satisfaction increased; effective under general anesthesia
Chanda & Levitin, 2013 [8]	Narrative review	Mechanistic synthesis	Music listening	Music engages reward/pleasure, stress/arousal, immunity, and social-affiliation neurochemical systems
Bringman et al., 2009 [9]	RCT	372 elective adults	Oral midazolam (0.05-0.1 mg/kg) vs relaxing music	Music produced greater STAI anxiety reduction than midazolam (p<0.001); no adverse effects
Kakar et al., 2021 [10]	SR & meta-analysis	20 RCTs; cardiac surgery	Recorded music, perioperative	Postoperative anxiety (SMD -0.50) and pain (-0.51) reduced; benefit persisted up to eight days
Kakde et al., 2023 [11]	RCT	108 parturients, elective cesarean delivery	Patient-selected music vs control during spinal anesthesia	Reduced postoperative anxiety (VAS-A MD -1.43) and pain catastrophizing; >95% rated satisfaction good/excellent
Dallı et al., 2022 [12]	RCT	36 ICU patients on mechanical ventilation	Music vs noise reduction vs control	Reduced delirium severity, pain, sedation need, and anxiety; fewer ventilator days in the music group
Oteri et al., 2021 [13]	Systematic review	27 studies; 2,558 brain-surgery patients	Multiple anxiety interventions (incl. music)	Preoperative anxiety prevalence was 17-89%; music, VR, acupuncture, and pharmacotherapy reduced anxiety
Jadavji-Mithani et al., 2015 [14]	RCT (qualitative)	29 awake craniotomy patients	Major-key vs minor-key music intraoperatively	Music was well tolerated; patients reported reduced anxiety before, during, and after; no adverse reactions
Azi et al., 2021 [15]	RCT	107 orthopedic patients under spinal anesthesia	Instrumental music vs no-music headphones	Reduced anxiety and intraoperative sedative requirement (p=0.004); 99% endorsed perioperative music
Tola et al., 2021 [16]	Systematic review	Breast cancer surgery patients	Music, aromatherapy, acupuncture, and massage	Music had a small-to-large effect on preoperative anxiety and a large effect on postoperative pain
van der Heijden et al., 2015 [17]	SR & meta-analysis	Pediatric surgery RCTs	Live or recorded music	Significant reductions in postoperative pain, anxiety, and distress
Muzzi et al., 2021 [18]	Four-arm RCT	104 children, adenotonsillectomy	Intraoperative music vs noise vs earplugs vs control	Music: large effect on pain on awakening; medium-to-large effect on emergence delirium
Li et al., 2024 [19]	SR & meta-analysis	19 RCTs	Perioperative music interventions	Reduced postoperative pain and anxiety on day one; attenuated blood pressure and heart rate fluctuations
Agüero-Millan et al., 2023 [20]	Umbrella review	17 SRs; 188 RCTs; 16,884 patients	Nonpharmacologic interventions for preoperative anxiety	Music is identified as the most studied and most consistently effective nonpharmacologic intervention
Petot et al., 2019 [21]	RCT protocol	Planned ≥170 gynecologic-surgery patients	Patient-selected vs predetermined music	Designed to test whether personalization adds incremental anxiolytic benefit

Personalized music as a component of precision anesthesia

Precision medicine has transformed oncology, genomics, and pharmacology. In anesthesia, the term precision has largely been applied to individualized drug dosing informed by pharmacogenomics, depth-of-anesthesia monitoring, and quantitative risk stratification. The concept of precision anesthesia is used here in a deliberately expanded sense: rather than redefining precision medicine in its pharmacologic or genomic form, the term is extended conceptually to propose that future models of precision anesthesia may benefit from incorporating individualized psychological and sensory preferences alongside these physiologic and pharmacologic factors. A technically flawless anesthetic may still be experienced as distressing if the patient's emotional and sensory needs are overlooked; conversely, attention to individualized comfort may improve trust, satisfaction, and the long-term perception of healthcare encounters [7,8,10,20].

A broader model for personalized anesthesia might include patient-selected music; individualized communication preferences; tailored anxiolytic strategies that minimize unnecessary benzodiazepine exposure; customized nausea-prevention regimens; environmental adjustments such as lighting and temperature; trauma-informed perioperative communication; and personalized emergence protocols. This approach recognizes emotional and psychological factors as clinically meaningful components of perioperative care rather than peripheral concerns. Personalized music selection may be notably valuable because it promotes a greater sense of autonomy and control during a period when patients often perceive a loss of control over their environment.

Certified registered nurse anesthetists (CRNAs) are ideally placed to contribute to this evolution. Through their roles in preoperative assessment, anesthetic planning, intraoperative management, induction, emergence, and patient advocacy, CRNAs frequently develop meaningful therapeutic relationships with patients throughout the surgical experience. Integrating individualized music preferences into perioperative workflows is closely aligned with the profession's longstanding emphasis on holistic, patient-centered anesthesia care.

Discussion

Comparison With Prior Reviews

Several major reviews have summarized the evidence base for perioperative music. The Cochrane review by Bradt and colleagues offered the most rigorous early synthesis of preoperative-anxiety trials and established music as a viable nonpharmacologic anxiolytic strategy [2]. The Lancet meta-analysis by Hole and colleagues expanded the scope to cover intraoperative and postoperative outcomes and quantified large effects on anxiety, pain, analgesic use, and satisfaction throughout diverse surgical populations [7]. Kakar and colleagues focused the evidence specifically on cardiac surgery, and the 2024 meta-analysis by Li and colleagues confirmed both anxiolytic and physiologic benefits on the first postoperative day [10,19]. The umbrella review by Aguero-Millan and colleagues, drawing on 17 systematic reviews, situated music among the wider landscape of nonpharmacologic interventions and identified it as the most consistently effective option [20].

These reviews focus primarily on aggregate effect estimates and on the question of whether music works. The present review builds upon this substantial body of evidence supporting perioperative music listening interventions and shifts the discussion toward implementation and personalization: how should the perioperative environment, and especially the practice of nurse anesthesia, be redesigned to deliver personalized music routinely as a component of precision anesthesia? In addition to a wider specialty synthesis (cardiac, obstetric, critical care, brain surgery, orthopedic, oncologic, and pediatric anesthesia) and the inclusion of a mechanistic neurochemical framework, this review contributes a practical perioperative workflow, an explicit discussion of personalization versus standardization, and a forward-looking integration with digital perioperative profiling and artificial intelligence. In doing so, the review attempts to bridge the gap between the favorable trial-level evidence summarized by prior reviews and the operational realities of routine anesthesia practice.

Music listening interventions should also be considered within the broader landscape of nonpharmacologic perioperative anxiety-management tools. Preoperative education, guided imagery, virtual reality, aromatherapy, acupuncture, hypnosis, and family presence have each demonstrated varying degrees of effectiveness in selected patient populations, though the quality and consistency of supporting evidence differ across interventions [13,16,18]. Unlike many of these alternatives, perioperative music listening is inexpensive, widely accessible, requires minimal staff training, and can be implemented across diverse perioperative settings with minimal equipment or workflow disruption. The umbrella review by Aguero-Millan and colleagues identified music as the most frequently studied and most consistently effective nonpharmacologic intervention for preoperative anxiety, supporting its potential role as a foundational component of patient-centered perioperative care [20]. Music may also be combined with, rather than viewed as a replacement for, these adjacent strategies and conventional pharmacologic anxiolysis.

A proposed clinical workflow for personalized perioperative music

Preoperative Assessment

During the preoperative anesthesia interview, patients should be invited to identify any music, sound, or audio content that helps them relax. A simple question, such as “Is there any music or sound that helps you relax before procedures?” can elicit useful preferences without adding substantial documentation time. Selected preferences should be recorded in the anesthesia preoperative assessment alongside other comfort and communication preferences.

Transport and Operating Room Integration

Music may be initiated during transport into the operating room and continued during monitor placement, intravenous access, and the placement of regional or neuraxial blocks. Volume should preserve clear team communication, patient safety, and the audibility of physiologic monitors. Personal devices, hospital-provided tablets, or shared streaming platforms may all be appropriate, depending on institutional infrastructure, privacy considerations, and infection-control policies.

Induction Phase

Music may continue until loss of consciousness with general anesthesia or until adequate sedation has been achieved during monitored anesthesia care or regional anesthesia. Given the evidence that music remains physiologically active under general anesthesia [7], continuing playback intraoperatively, when feasible and acceptable to the operating team, may also be considered.

Emergence Phase

In selected patients, particularly children and adults with a history of emergence agitation or postoperative delirium, familiar music played during emergence may reduce agitation and improve orientation [18]. A patient-specific “emergence playlist” may be incorporated into the anesthetic plan alongside other strategies such as alpha-2 agonist administration and quiet, low-stimulus environments.

The role of artificial intelligence and digital perioperative profiling

The following discussion is forward-looking and is presented as a research and implementation agenda rather than as established evidence. Perioperative information systems may progressively integrate patient preference profiles directly into anesthesia workflows. Potential components include validated anxiety-profiling algorithms, personalized environmental settings, artificial-intelligence-assisted sedation prediction, integration of music preferences into the anesthetic record, predictive emergence-management tools, and adaptive communication recommendations customized to a patient's prior healthcare experiences. Patients may eventually complete digital preoperative preference questionnaires (covering music, communication method, sensory preferences, and trauma history) prior to surgery, allowing anesthesia teams to individualize care before the day of the operation.

Artificial intelligence systems may also help identify patients at elevated risk for perioperative anxiety, persistent postoperative pain, or emergence delirium, prompting evidence-based emotional-support interventions in addition to pharmacologic strategies [1,18]. The convergence of personalized music, digital perioperative profiling, and AI-based risk stratification represents a plausible near-term frontier for nurse anesthesia practice, though each component will require prospective validation before routine clinical integration.

Challenges and limitations

Despite its promise, the implementation of personalized perioperative music presents several operational considerations. Infection-control policies must address personal devices, headphones, and shared audio equipment. Operating room communication must be preserved, and music volume should never compromise team-based safety practices or the audibility of alarms and monitors. Surgeon and staff preferences vary, and institutional buy-in is essential for sustainable adoption. Streaming connectivity, copyright and licensing considerations, and equipment ownership must also be addressed at the institutional level.

Patient privacy, consent, and information security are additional considerations. Institutions implementing personalized music programs should ensure that personal information, account details, messages, and notifications remain protected and suppressed during clinical use, and that personal streaming accounts are managed in compliance with applicable privacy and information-security policies. Patient consent for the use of personal devices and accounts in the perioperative environment should be obtained as part of the broader anesthesia consent discussion. Music preferences are highly individualized, and some patients may prefer silence or may find certain forms of auditory stimulation distracting rather than comforting; the offer of music must therefore remain optional and patient-directed.

This narrative review also has research limitations that justify a measured interpretation of its conclusions. As a narrative rather than a systematic review, it is susceptible to selection bias in the identification and inclusion of studies, and it did not generate pooled effect-size estimates or a PRISMA flow diagram. It reflects a single-author appraisal, did not incorporate a formal quantitative risk-of-bias assessment (such as Cochrane RoB 2, ROBINS-I, or GRADE), and was restricted to English-language publications. Much of the existing evidence relies on heterogeneous interventions, varying music selections, and inconsistent outcome measures, and most randomized trials have evaluated generalized relaxing music rather than truly patient-selected music. Several of the pooled meta-analyses reported substantial statistical heterogeneity, and the included trials varied in blinding, allocation concealment, and outcome ascertainment, so pooled effect estimates should be regarded as approximate rather than precise. Many of the reported relationships among anxiety, music exposure, and clinical outcomes are observational and should be interpreted as associations rather than as established causal effects. Publication bias may also contribute to an overrepresentation of positive findings within the perioperative music literature. Additional rigorous trials are required to quantify the incremental benefit of personalization, characterize the populations most likely to benefit, and assess the durability of effects across surgical specialties [2,20,21].

Future directions

Future research needs to evaluate personalized versus standardized music listening interventions in head-to-head randomized trials; quantify sedative- and opioid-sparing effects across surgical populations; assess impact on postoperative recovery metrics including time to discharge and return-to-baseline function; investigate effects on emergence delirium and postoperative cognitive outcomes; measure patient-reported satisfaction and trust; integrate personalized music into enhanced-recovery-after-surgery (ERAS) pathways; and pilot AI-assisted personalization systems within the electronic anesthesia record. Future randomized studies should specifically determine whether personalization itself provides incremental benefit beyond the established effects of music listening interventions alone, which remains the central unanswered scientific question in this area.

Future investigations should also evaluate implementation feasibility, provider acceptance, cost-effectiveness, workflow integration, and potential differences across age groups, cultural backgrounds, and surgical specialties. Human-centered anesthesia care may ultimately become a distinguishing feature of high-quality perioperative medicine, with measurable effects on clinical outcomes and the lived experience of surgical patients.

## Conclusions

Personalized perioperative music listening represents more than a comfort measure. It may signal the beginning of a wider transformation toward emotionally intelligent, patient-centered anesthesia care. As anesthesia practice continues to evolve technologically, providers may also benefit from recognizing the importance of individualized psychological and sensory experiences in perioperative medicine. Future models of precision anesthesia, understood as a conceptual extension beyond physiologic and pharmacologic optimization, may benefit from incorporating personalization of the human experience itself.

Incorporating patient-selected music into perioperative workflows offers a low-cost, scalable, and evidence-supported intervention with the potential to reduce anxiety, decrease pharmacologic anxiolytic requirements, improve patient satisfaction, and strengthen the therapeutic relationship between patients and anesthesia providers. The integration of individualized sensory preferences may represent a meaningful step toward more patient-centered perioperative care, pending confirmation in adequately powered, personalization-focused trials.

## References

[1] Friedrich S, Reis S, Meybohm P, Kranke P (2022). Preoperative anxiety. Curr Opin Anaesthesiol.

[2] Bradt J, Dileo C, Shim M (2013). Music interventions for preoperative anxiety. Cochrane Database Syst Rev.

[3] Spielberger CD, Gorsuch RL, Lushene RE, Vagg PR, Jacobs GA (1983). Manual for the State-Trait Anxiety Inventory (Form Y1 - Y2). https://www.researchgate.net/publication/235361542_Manual_for_the_State-Trait_Anxiety_Inventory_Form_Y1_-_Y2.

[4] Kindler CH, Harms C, Amsler F, Ihde-Scholl T, Scheidegger D (2000). The visual analog scale allows effective measurement of preoperative anxiety and detection of patients' anesthetic concerns. Anesth Analg.

[5] Moerman N, van Dam FS, Muller MJ, Oosting H (1996). The Amsterdam Preoperative Anxiety and Information Scale (APAIS). Anesth Analg.

[6] Sullivan MJL, Bishop SR, Pivik J (1995). The Pain Catastrophizing Scale: development and validation. Psychol Assess.

[7] Hole J, Hirsch M, Ball E, Meads C (2015). Music as an aid for postoperative recovery in adults: a systematic review and meta-analysis. Lancet.

[8] Chanda ML, Levitin DJ (2013). The neurochemistry of music. Trends Cogn Sci.

[9] Bringman H, Giesecke K, Thörne A, Bringman S (2009). Relaxing music as pre-medication before surgery: a randomised controlled trial. Acta Anaesthesiol Scand.

[10] Kakar E, Billar RJ, van Rosmalen J, Klimek M, Takkenberg JJ, Jeekel J (2021). Music intervention to relieve anxiety and pain in adults undergoing cardiac surgery: a systematic review and meta-analysis. Open Heart.

[11] Kakde A, Lim MJ, Shen H, Tan HS, Tan CW, Sultana R, Sng BL (2023). Effect of music listening on perioperative anxiety, acute pain and pain catastrophizing in women undergoing elective cesarean delivery: a randomized controlled trial. BMC Anesthesiol.

[12] Dallı ÖE, Yıldırım Y, Aykar FŞ, Kahveci F (2023). The effect of music on delirium, pain, sedation and anxiety in patients receiving mechanical ventilation in the intensive care unit. Intensive Crit Care Nurs.

[13] Oteri V, Martinelli A, Crivellaro E, Gigli F (2021). The impact of preoperative anxiety on patients undergoing brain surgery: a systematic review. Neurosurg Rev.

[14] Jadavji-Mithani R, Venkatraghavan L, Bernstein M (2015). Music is beneficial for awake craniotomy patients: a qualitative study. Can J Neurol Sci.

[15] Azi LM, Azi ML, Viana MM, Panont AL, Oliveira RM, Sadigursky D, Alencar DF (2021). Benefits of intraoperative music on orthopedic surgeries under spinal anesthesia: a randomized clinical trial. Complement Ther Med.

[16] Tola YO, Chow KM, Liang W (2021). Effects of non-pharmacological interventions on preoperative anxiety and postoperative pain in patients undergoing breast cancer surgery: a systematic review. J Clin Nurs.

[17] van der Heijden MJ, Oliai Araghi S, van Dijk M, Jeekel J, Hunink MG (2015). The effects of perioperative music interventions in pediatric surgery: a systematic review and meta-analysis of randomized controlled trials. PLoS One.

[18] Muzzi E, Ronfani L, Bossini B, Lezcano C, Orzan E, Barbi E (2021). Effects of intraoperative auditory stimulation on pain and agitation on awakening after pediatric adenotonsillectomy: a randomized clinical trial. JAMA Otolaryngol Head Neck Surg.

[19] Li G, Yu L, Yang Y, Deng J, Shao L, Zeng C (2024). Effects of perioperative music therapy on patients with postoperative pain and anxiety: a systematic review and meta-analysis. J Integr Complement Med.

[20] Agüero-Millan B, Abajas-Bustillo R, Ortego-Maté C (2023). Efficacy of nonpharmacologic interventions in preoperative anxiety: a systematic review of systematic reviews. J Clin Nurs.

[21] Petot T, Bouscaren N, Maillard O, Huiart L, Boukerrou M, Reynaud D (2019). Comparing the effects of self-selected music versus predetermined music on patient anxiety prior to gynaecological surgery: a study protocol for a randomised controlled trial. Trials.

